# Exposure to specific polyfluoroalkyl chemicals is associated with cardiovascular disease in US adults: a population-based study

**DOI:** 10.3389/fcvm.2024.1487956

**Published:** 2025-01-09

**Authors:** Wenwen Xiao, Guojin Jian, Fei Ma, Hong Li, Xiaohong Yang, Hengyang Zhang, Yongping Cao

**Affiliations:** ^1^ Eastern Theater Command Centers for Disease Control and Prevention, Nanjing, China; ^2^ Department of Cardiology, PLA Joint Logistic Support Force 902 Hospital, Bengbu, China

**Keywords:** polyfluoroalkyl chemicals (PFCs), cardiovascular disease (CVD), national health and nutrition examination survey (NHANES), mixed exposure, risk factors

## Abstract

**Background:**

Polyfluoroalkyl chemicals (PFCs) present potential health risks due to their persistence and bioaccumulation. However, there is currently insufficient evidence regarding their impact on cardiovascular disease (CVD). Consequently, it is imperative to investigate the correlation between PFCs and CVD.

**Methods:**

The data was collected from National Health and Nutrition Examination Survey in 2005–2012. Logistic regression models were employed to assess the association between single PFC and CVD. Generalized additive model (GAM) was used for evaluating nonlinear relationships. Subgroup analyses were conducted to explore interaction effects. Bayesian kernel machine regression (BKMR) and weighted quantile sum (WQS) models were used to evaluate the joint effect of PFC exposures on CVD.

**Results:**

In logistic regression, PFDE, MPAH, and PFUA were positively associated with CVD. In the GAM, there was a significant nonlinear relationship between MPAH and CVD. Subgroup analysis revealed the interaction of gender and race in the effects of PFCs and CVD. PFUA was positively correlated with CVD in males but show no significant difference in females. PFDE was positively associated with CVD among non-Hispanic white individual. The results of BKMR indicated that the impact of mixed PFCs on CVD increased initially and then weakened, showing an overall positive trend. The results of WQS suggested that PFDO contributed most to the effect.

**Conclusion:**

Our study showed that serum PFDE, MPAH, and PFUA levels were positively correlated with CVD. PUFA was found to interact with gender and race in relation to CVD. A general positive correlation exists between mixed exposure to PFCs and CVD, with PFDO being the most contributory PFC. Our study provided important evidence for probing the impact of PFCs on CVD and laid a foundation for further mechanism research.

## 1 Introduction

Cardiovascular disease (CVD) is a disease of heart and blood vessels, commonly including stroke, coronary heart disease, congestive heart failure, among others (1–3). While CVD caused no more than 10 percent of all deaths in the last century, it accounted for 31 percent of deaths in 2016, surpassing any other disease (4). CVD poses a global burden, especially in middle-income and high-income countries. The prevalence of CVD is also increasing annually even in some lower-income countries (5). Despite protective measures aimed at reducing the incidence of CVD, millions of individuals still die from it each year (6). Consequently, CVD has become a pressing global public health issue, with an impact that cannot be ignored.

Risk factors such as unhealthy diets, insufficient exercise, tobacco use and alcohol abuse have been linked to heart disease and stroke (7–12). Harmful behavioral factors can lead to evaluated blood pressure, blood glucose, blood lipids and overweight in individuals, all of which can increase the likelihood of CVD (13, 14). Additionally, numerous studies have indicated that environmental chemicals play a significant role in the development of CVD, highlighting the urgent need to determine whether certain environmental factors affect CVD (15).

Polyfluoroalkyl chemicals (PFCs) are a class of synthetic organic compounds where the hydrogen atoms in the alkyl group are replaced by some or all of the fluorine atoms (16). PFCs are widely used in various industrial and consumer applications, such as manufacturing coatings, waterproof materials, food packaging, and cleaning agents, due to their excellent stability and corrosion resistance (17, 18). FPCs exhibit strong environmental persistence and can be detected around the world (19). As persistent organic pollution, FPCs can accumulate in the human body through drinking water, ingestion and other routes, causing significant health damage (20, 21). Numerous studies have shown that exposure to PFCs is associated with adverse health outcomes, including cancer, immune system dysfunction, reproductive disorders, and thyroid dysfunction (22–25). These findings have generated global concern and highlighted the importance of monitoring and regulating the use of PFCs to mitigate their impacts on human health and the environment.

For human exposure assessment, internal exposure can provide more realistic and accurate results due to the direct detection of pollutant concentrations in human tissues or body fluids. Serum PFC levels become a crucial biomarker for assessing their influence on cardiovascular health. The past studies have often focused on the effect of a single PFC. In fact, mixed exposures tend to be closer to real human exposure levels. In addition, there may be interactions between PFCs that have the potential to produce more severe health damage. Therefore, evaluating the combined effect of PFCs on CVD is considered necessary. The study aims to assess the potential association between single and combined PFCs and CVD from NHANES in 2005–2012.

## 2 Material and methods

### 2.1 Study population

NHANES is a nationwide cross-sectional study utilizing a complex probability sampling design. It collects a variety of information from different groups of participants. The aim of NHANES is to assess the health and nutritional status of both children and adults in the US. The program started in the 1960s and has been a continuing program since 1999, focusing on a range of health and nutrition issues. The project investigates a nationally representative sample of approximately 5,000 people each year. The data is published every two years, allowing public researchers to obtain valuable insights.

**Figure 1:**
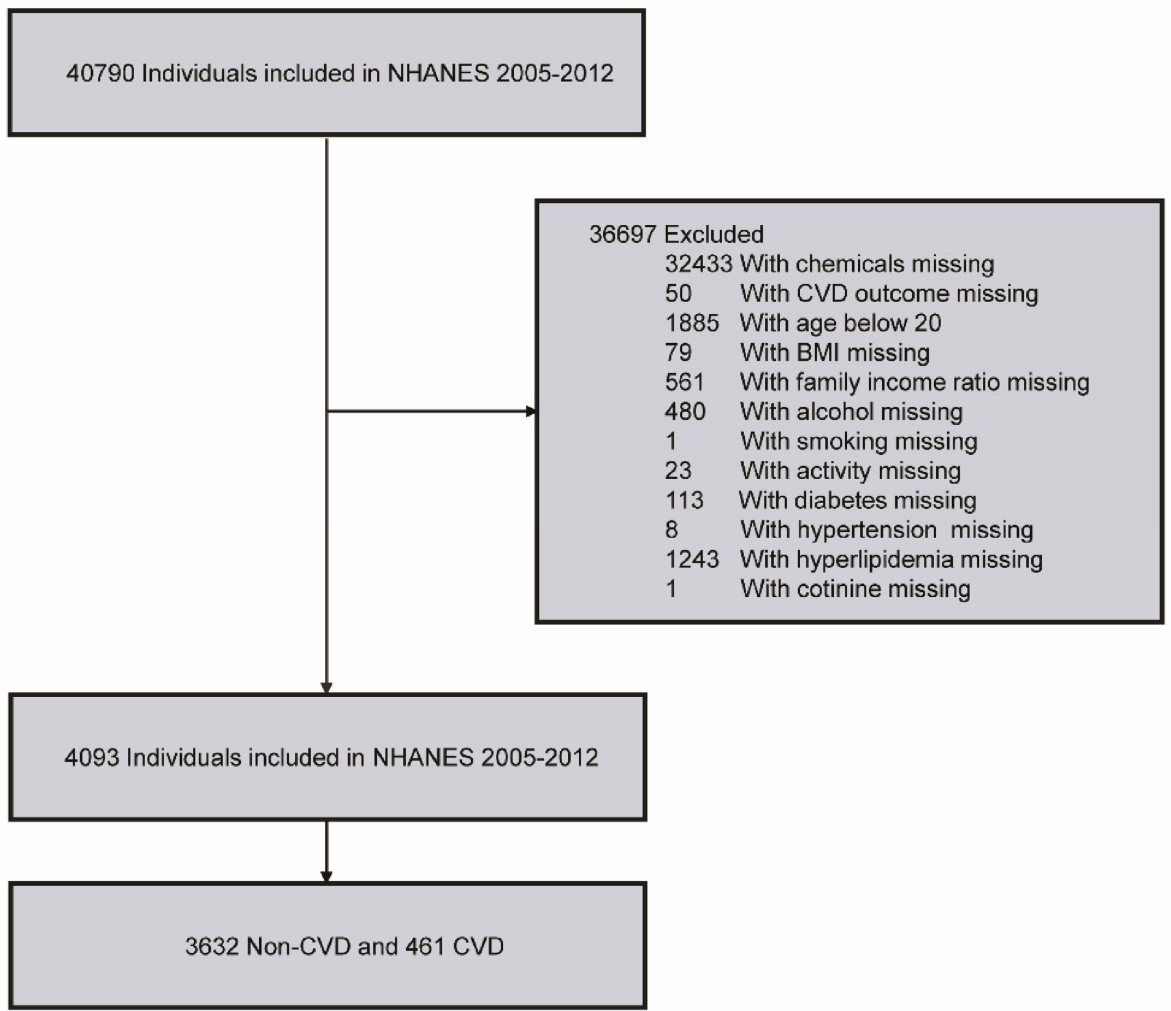
Flowchart of inclusion and exclusion criteria for our study.

In this study, the data was collected from NHANES in 2005–2012. Initially, a total of 40,790 participants were identified. Then, 32,433 participants with missing PFC exposure values and 50 participants with missing CVD outcome were excluded. Participants were further included or excluded based on the following criteria: (1) demographic factors (gender, age ≥ 20, race, education, family income ratio); (2) lifestyle indicators (smoking status, alcohol consumption, physical activity); (3) disease history (hypertension, hyperlipidemia, diabetes); and (4) other indicators [body mass index (BMI), cotinine]. After applying these exclusion criteria, the final study cohort comprised 4,093 participants (Figure 1).

### 2.2 Variables and outcome assessment

This study focused on PFCs as the independent variables of interest. Spot serum samples were collected at mobile examination centers (MECs) and processed, stored, shipped to the Division of Laboratory Sciences, National Center for Environmental Health, Centers for Disease Control and Prevention for analysis. Serum PFC concentrations were quantitatively measured using online solid phase extraction, high-performance liquid chromatography-tandem ionization and tandem mass spectrometry. Briefly, serum samples were diluted with formic acid and injected into a commercial column switching system to concentrate analytes onto a solid phase extraction column. The analytes were then separated from other serum components by high-performance liquid chromatography. The negative ion TurboIonSpray ionization method was employed for detection and quantification, with a detection limit (LOD) of 0.1 ng/ml. Results below the LOD were recorded as the square root of LOD divided by 2.

Various covariates were included into the analysis, such as gender, age, race, education level, family income ratio, hypertension, hyperlipidemia, diabetes, alcohol consumption, smoking status, physical activity, BMI and cotinine levels.

CVD outcomes were derived from structured questionnaire files. Specific types include congestive heart failure, coronary heart disease, angina, heart attack, and stroke. Participants answering “yes” to relevant medical history questions were classified as having a history of CVD, while those answering “no” were categorized as not having a history of CVD.

### 2.3 Statistical analysis

Descriptive statistics was conducted for all variables. Continuous variables were presented as mean ± standard deviation. Categorical variables were expressed as a count and percentage. Demographic and health-related characteristics were compared between participants with and without CVD using *t*-tests for continuous variables. Chi-square tests were used for categorical variables. Logarithmic transformation was applied for variables that did not follow a normal distribution.

Multivariable logistic regression models were used to evaluate the association between single PFC and CVD. Model 1 involved univariate logistic regression analysis. Model 2 adjusted for demographic variables including age, gender, race, education level, and family income ratio. Model 3 further adjusted for lifestyle factors, medical history and physical indicators such as smoking, alcohol consumption, physical activity, diabetes, hypertension, hyperlipidemia, BMI, and serum cotinine. Subgroup analyses were employed to explore interactions between single PFC and other covariates.

The generalized additive model (GAM) was employed to examine potential nonlinear relationships, utilizing a 3-degree-of-freedom natural cubic spline for smoothing and reviewing dose-response curves.

Considering the common mixed exposure scenarios in daily life, evaluating combined PFC exposure was crucial. The Bayesian kernel machine regression (BKMR) model was used to evaluate the effect of mixed PFC exposure on CVD. The analytical method calculated the potential continuous outcome change of CVD with PFCs exposure ranging from the 25th to the 75th percentile and estimated the exposure-response function between individual PFCs exposure and CVD. The weighted quantile sum (WQS) model was utilized to assess the impact of combined exposure to PFCs and evaluate the relative contribution of each chemical.

Sensitivity analysis was performed by changing the adjustment covariates in models 1, 2, and 3 to assess the robustness of the results. Model 1 remained unadjusted. Model 2 incorporates adjustments for age, gender, race, education level, and family income ratio. Model3 further adjusted for alcohol consumption, smoking status, physical activity, hypertension, hyperlipidemia, diabetes, BMI, and cotinine levels.

All analyses were performed using R software (version 4.3.2). A *P*-value of less than 0.05 was considered statistically significant.

## 3 Results

### 3.1 Baseline characteristics

General information about the population of the study was presented in Table 1. 4,093 participants were included in the final analysis of the relationship between PFCs and CVD. Among the participants, 461 were identified as CVD patients, while 3,632 were non-CVD patients. The average age of participants was 52.01 ± 17.27 (mean ± SD). Compared to non-CVD participants, those with CVD were observed to be older, more frequently male, and of non-Hispanic white ethnicity. CVD patients had a higher proportion of education level above high school and were more prone to suffer from hypertension, hyperlipidemia, and diabetes. Furthermore, individuals with CVD had a higher smoking rate, relatively higher BMI, elevated serum cotinine levels. Compared to non-CVD patients, a lower proportion of CVD patients engaged in physical activities.

**Table 1:** Characteristics of the study population.

Characteristics	Non-CVD (*N* = 3,632)	CVD (*N* = 461)	*P* value
Age, years	50.09 (16.84)	67.11 (12.48)	<0.01
Gender			<0.01
Male	1,696 (46.7)	285 (51.8)	
Female	1,936 (53.3)	176 (38.2)	
Race			<0.01
Mexican American	454 (12.5)	40 (8.7)	
Other Hispanic	308 (8.5)	29 (6.3)	
Non-Hispanic White	1,840 (50.7)	289 (62.7)	
Non-Hispanic Black	743 (20.5)	86 (18.7)	
Other Race	287 (7.9)	17 (3.7)	
Education			<0.01
Less than high school	768 (21.1)	173 (37.5)	
High school or equivalent	755 (20.8)	107 (23.2)	
College or above	2,107 (58.0)	180 (39.0)	
Other	2 (0.1)	1 (0.2)	
Family income ratio			<0.01
≤1.30	931 (25.6)	158 (34.3)	
1.31–3.50	1,344 (37.0)	184 (39.9	
>3.5	1,357 (37.4)	119 (25.8)	
Hypertension			<0.01
Yes	1,270 (35.0)	350 (75.9)	
No	2,362 (65.0)	111 (24.1)	
Hyperlipidemia			<0.01
Yes	1,387 (38.2)	300 (65.1)	
No	2,245 (61.8)	161 (34.9)	
Diabetes			<0.01
Yes	410 (11.3)	145 (31.5)	
No	3,222 (88.7)	316 (68.5)	
Alcohol			0.29
Yes	2,611 (71.9)	220 (69.4)	
No	1,021 (28.1)	141 (30.6)	
Smoke			<0.01
Yes	1,556 (42.8)	292 (63.3)	
No	2,076 (57.2)	169 (36.7)	
Activities			<0.01
Yes	977 (26.9)	73 (15.8)	
No	2,655 (73.1)	388 (84.2)	
BMI (kg/m^2^)			0.01
<25	1,054 (29.0)	108 (23.4)	
≥25	2,578 (71.0)	353 (76.6)	
Cotinine(ng/ml)	49.36 (122.66)	75.57 (144.61)	<0.01

Data (unweighted) are presented as mean (SD) or *n* (%); For categorical variables, *P* values were analyzed by chi-square tests. For continuous variables, *P* values were analyzed by the *t*-test.

### 3.2 Characteristics of PFCs in serum

The detection rates and detection limits of the 10 PFCs analyzed in serum were shown in Supplementary Table S1. PFOA (99.68%), PFOS (99.73%) and PFNA (99.43%) were the three PFCs with the highest detection rate. There are four types of PFCs with a detection rate below 70%, which are PFHP (12.73%), PFSA (7.180%), PFDO (5.70%) and PFUA (54.70%). The distribution of serum PFCs in CVD and non-CVD patients were presented in Supplementary Table S2. Serum concentrations of PFOA, PFOS, PFHS, PFSA, and PFUA in CVD group were significantly higher than those in non-CVD group (*P* < 0.05). The spearman's correlation coefficient ranged from 0.02 to 0.77, indicating different correlations among PFCs (Supplementary Figure S1).

### 3.3 Association analysis between PFCs and CVD

The linear association between PFCs and CVD was assessed using multiple logistic regression analyses, as summarized in Table 2. After adjusting for demographic covariates, our analysis revealed a positive correlation between MPAH and PFUA levels and CVD incidence (OR, 1.14; 95% CI, 1.02–1.27; *P* = 0.02; OR, 1.14; 95% CI, 1.00–1.31; *P* = 0.05; OR). After adjusting for all potential confounders, elevated serum concentrations of PFDE, MPAH, and PFUA were associated with increased incidence of CVD (OR, 1.17; 95% CI, 1.02–1.34; *P* = 0.03; OR, 1.15; 95% CI, 1.03–1.29; *P* = 0.01; OR, 1.21; 95% CI, 1.05–1.39; *P* < 0.01). However, no significant associations were observed for other PFCs.

**Table 2:** Logistic regression associations of PFCs with CVD in adults.

PFCs	Model 1			Model 2			Model 3		
	OR	95% CI	*P* value	OR	95% CI	*P* value	OR	95% CI	*P* value
PFOA	1.25	1.09–1.44	<0.01**	0.95	0.82–1.10	0.51	0.96	0.82–1.11	0.57
PFOS	1.45	1.30–1.63	<0.01**	0.99	0.88–1.11	0.83	1.02	0.90–1.15	0.77
PFDE	1.23	1.09–1.39	<0.01**	1.13	0.99–1.29	0.08	1.17	1.02–1.34	**0**.03*
PFHS	1.19	1.07–1.32	<0.01**	0.90	0.79–1.01	0.08	0.9	0.79–1.02	0.09
MPAH	1.51	1.37–1.66	<0.01**	1.14	1.02–1.27	**0**.02*	1.15	1.03–1.29	**0**.01*
PFNA	1.29	1.12–1.49	<0.01**	1.11	0.96–1.30	0.16	1.09	0.94–1.28	0.25
PFHP	1.22	1.07–1.39	<0.01**	1.06	0.92–1.22	0.45	1.05	0.91–1.22	0.49
PFSA	1.69	1.30–2.15	<0.01**	1.13	0.84–1.50	0.40	1.26	0.93–1.69	0.12
PFDO	1.36	1.08–1.72	<0.01**	1.18	0.91–1.53	0.22	1.25	0.95–1.64	0.10
PFUA	1.25	1.11–1.40	<0.01**	1.14	1.00–1.31	**0**.05*	1.21	1.05–1.39	**<0**.01**

Model 1 was univariate logistic regression; Model 2 was adjusted for gender, age, race, education, family income ratio; Model 3 was adjusted for gender, age, race, education, family income ratio, smoking, drinking, physical activity, diabetes, hypertension, hyperlipidemia, BMI and cotinine.

Bold highlights PFCs with positive results.

* *P* < 0.05.

** *P* < 0.01.

**Figure 2:**
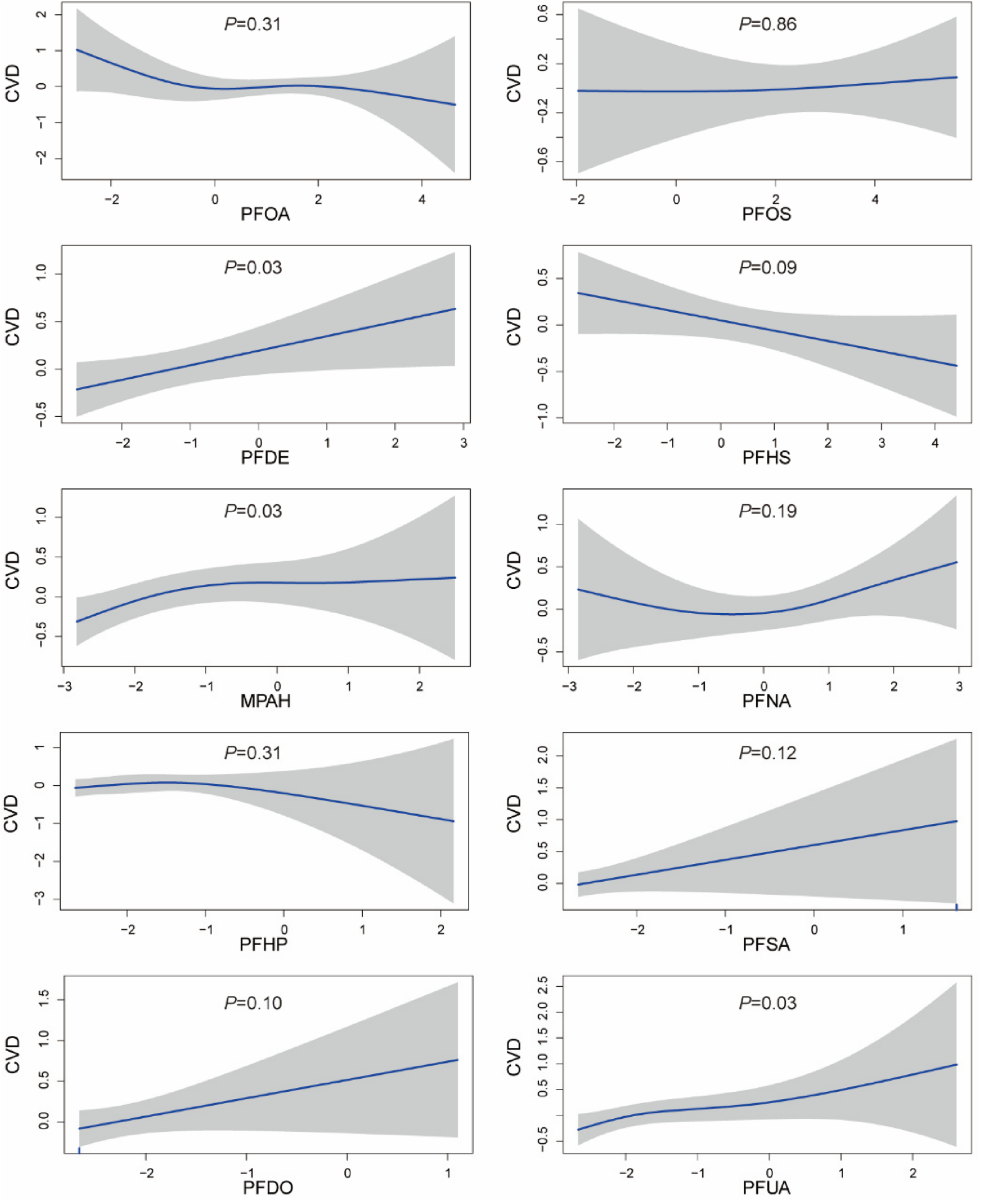
Generalized additive model plot of the association between PFCs and CVD. The model was adjusted for age, gender, race, education levels, family income ratio, alcohol, smoking, activities, hypertension, hyperlipidemia, diabetes, BMI, and cotinine.

The GAM was employed to elucidate the nonlinear association between PFCs and CVD, as depicted in Figure 2. The analysis revealed a notable non-linear correlation between MPAH and CVD (*P* < 0.05). Although PFDE and PFUA exhibited statistical differences, visual observation showed a linear correlation Furthermore, visual examination suggested non-linear associations between PFOA and PFNA with CVD; however, statistical tests did not provide evidence of significant differences.

### 3.4 Stratified association between PFCs and CVD

Our analysis unveiled an interaction effect between PFUA exposure and gender (Table 3). Specifically, a significant positive association was found between PFUA and CVD in male (OR, 1.34, 95% CI, 1.12–1.60, *P* < 0.01), while no significant association was observed in female. Other PFCs did not demonstrate interaction effects with gender. Significant interactions with race were observed for PFOA, PFDE, PFNA and PFUA (*P* = 0.04, *P* < 0.01, *P* = 0.04, *P* < 0.01). PFDE was positively linked to CVD in non-Hispanic White (OR, 1.37, 95% CI, 1.14–1.65, *P* < 0.01). PFUA was positively associated with CVD among non-Hispanic whites (OR, 1.41; 95% CI, 1.17–1.71, *P* < 0.01). Despite observing an interaction between PFOA, PFNA and race, with a positive association in Hispanic individuals and a negative correlation in other races, the disparity was not statistically significant. The results of the stratified analysis of the other covariates were described in Supplementary Table S3–S11.

**Table 3:** Stratified association between PFCs and CVD.

PFCs	Gender							Race						
	Male			Female				Non-Hispanic White			Other			
	OR	95% CL	*P* value	OR	95% CL	*P* value	P-int	OR	95% CL	*P* value	OR	95% CL	P value	*P*-int
PFOA	0.97	0.79–1.20	0.78	0.95	0.45–1.20	0.64	0.51	1.08	0.88–1.34	0.46	0.86	0.69–1.07	0.16	**0**.04*
PFOS	1.08	0.91–1.27	0.4	0.95	0.79–1.15	0.61	0.08	1.04	0.88–1.24	0.61	1	0.84–1.20	0.98	0.21
PFDE	1.22	1.02–1.46	**0**.03*	1.09	0.87–1.35	0.45	0.28	1.37	1.14–1.65	**<0**.01**	0.95	0.77–1.17	0.64	**<0**.01**
PFHS	0.89	0.75–1.06	0.2	0.94	0.78–1.14	0.54	0.67	0.93	0.78–1.11	0.41	0.88	0.73–1.07	0.2	0.26
MPAH	1.13	0.97–1.31	0.12	1.18	0.99–1.41	0.06	0.39	1.07	0.92–1.24	0.36	1.28	1.08–1.52	<0.01	0.34
PFNA	1.14	0.93–1.41	0.2	1.04	0.83–1.32	0.71	0.52	1.22	1–1.49	0.06	0.95	0.75–1.20	0.66	**0**.04*
PFHP	1	0.82–1.22	0.98	1.09	0.86–1.37	0.49	0.84	1.05	0.87–1.28	0.59	1.08	0.85–1.38	0.51	0.96
PFSA	1.19	0.83–1.68	0.34	1.29	0.65–2.27	0.42	0.69	1.19	0.85–1.65	0.31	1.44	0.69–2.64	0.27	0.72
PFDO	1.24	0.86–1.77	0.25	1.23	0.80–1.85	0.34	0.59	1.31	0.90–1.89	0.15	1.23	0.82–1.84	0.31	0.74
PFUA	1.34	1.12–1.60	**<0**.01**	1	0.79–1.26	0.99	**0**.03*	1.41	1.17–1.71	**<0**.01**	1.01	0.83–1.22	0.95	**<0**.01**

Analyses were adjusted for gender, age, race, education level, family income ratio, smoking, drinking, physical activity, diabetes, hypertension, hyperlipidemia, BMI, and cotinine.

Bold highlights PFCs with positive results.

* *P* < 0.05.

** *P* < 0.01.

### 3.5 Effects of mixed exposure of PFCs on CVD

**Figure 3:**
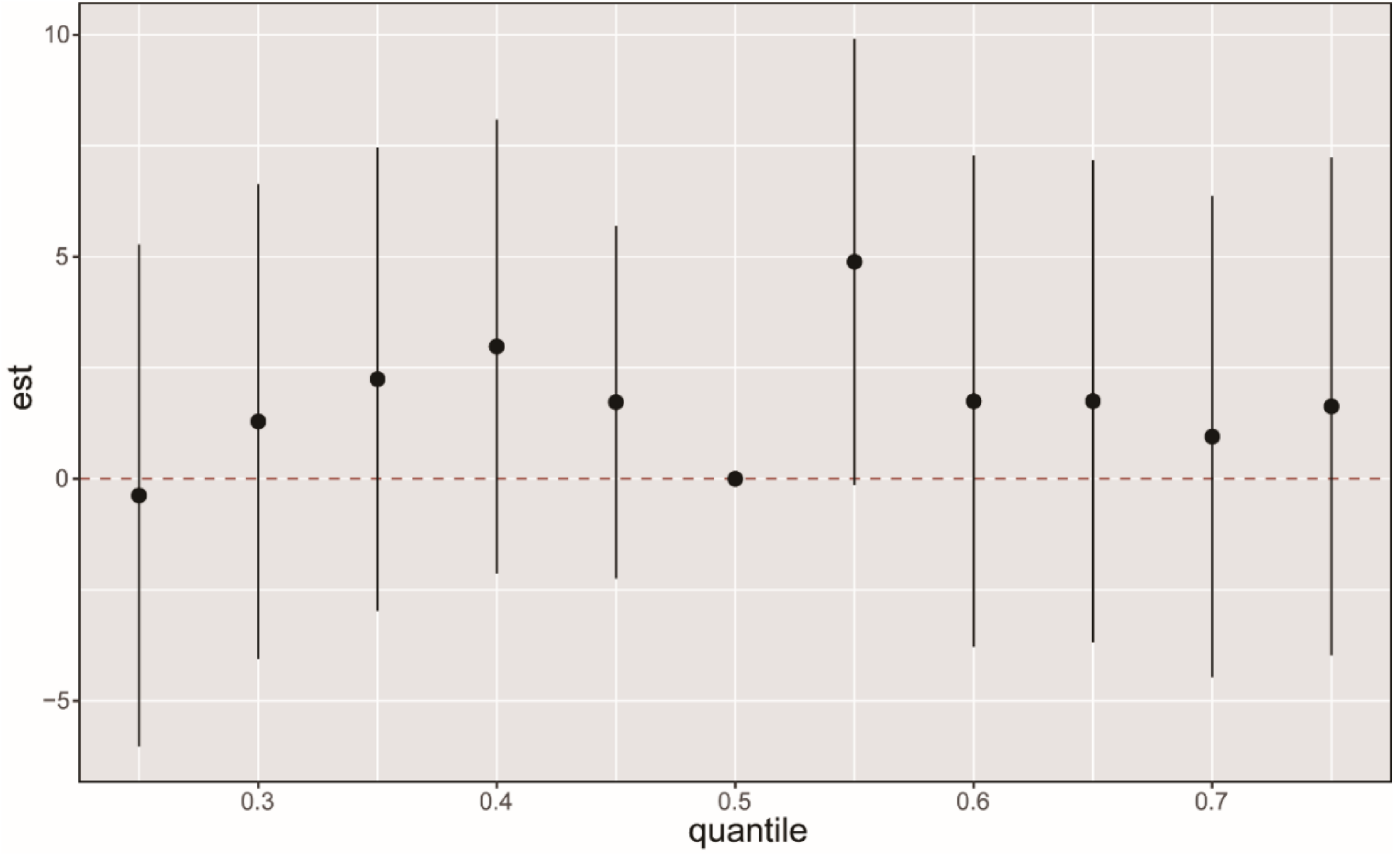
BKMR analysis on the FPCs mixed exposure and CVD. The BKMR model allows US to estimate the overall effect (95% CI) of the PFC mixture on CVD when comparing all chemicals in a specific percentile to those in the 50th percentile. The “est” parameter can be interpreted as the relationship between chemicals and potential continuous outcomes, which serve as continuous markers of binary CVD outcomes. The results were adjusted for age, gender, race, educational levels, family income ratio, alcohol, smoking, activities, hypertension, hyperlipidemia, diabetes, BMI, and cotinine.

**Figure 4:**
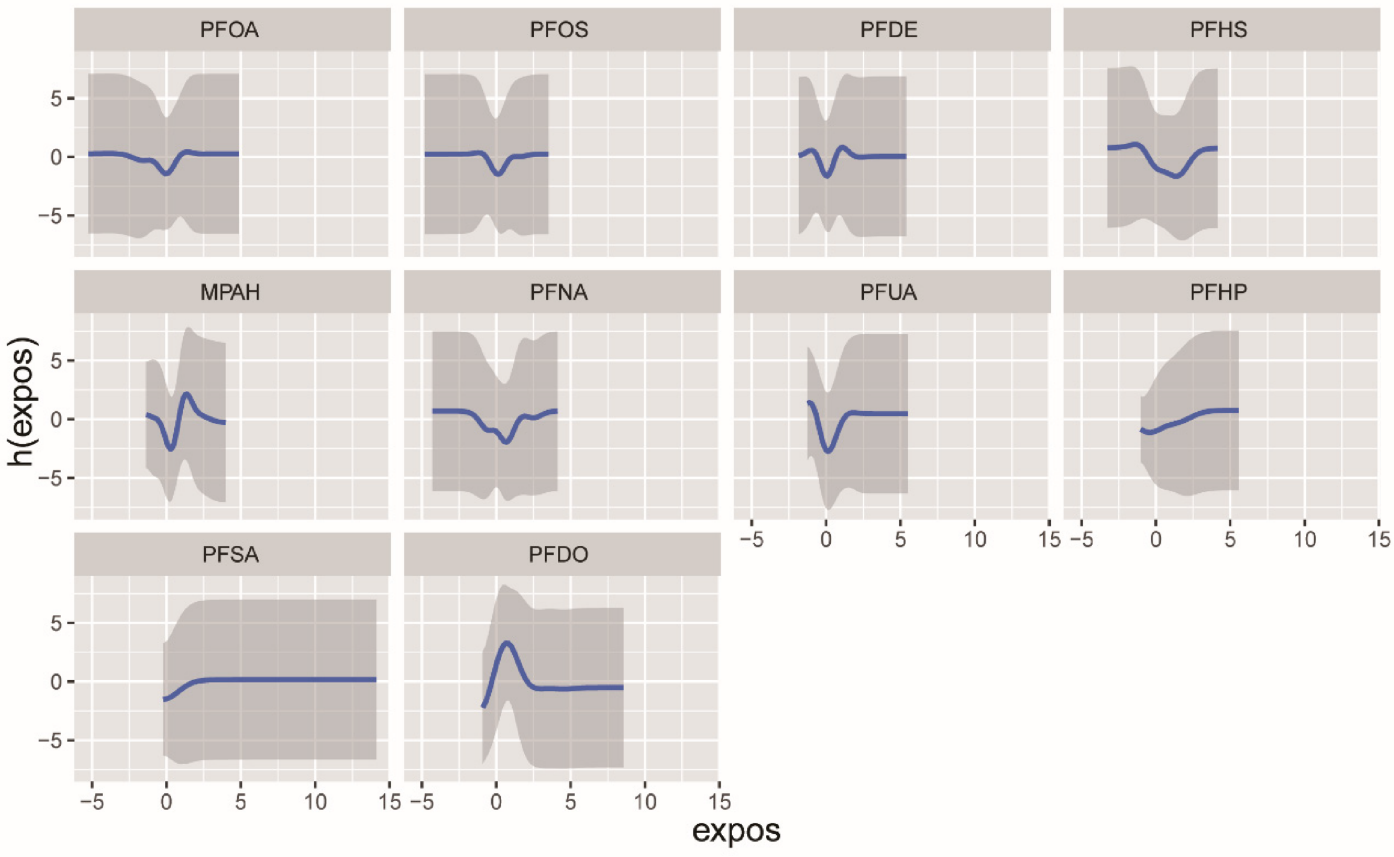
The univariate exposure-response function (95% CI) between the concentration of selected chemicals and CVD was determined while keeping the concentration of other chemicals at the median. The function h(expos) represents the relationship between chemicals and potential continuous outcomes, which serve as continuous markers of binary obesity outcomes. The results were adjusted for age, gender, race, educational levels, family income ratio, alcohol, smoking, activities, hypertension, hyperlipidemia, diabetes, BMI and cotinine.

We employed the BKMR model to examine the impact of combined PFCs exposure on CVD. The overall effect of PFCs on CVD tends to exhibit an initial increase followed by a decrease, indicating an overall promoting effect (Figure 3). While we found a positive correlation between PFOA, PFDO, and CVD at the 0.25th and 0.5–0.75 quantiles, the results were not statistically significant (Supplementary Figure S2). Examining the exposure-response correlation of individual PFCs, we observed that PFOA, PFOS, PFHS, PFNA, and PFUA displayed a pattern of initial decrease followed by an increase in the dose-response curve, whereas PFDO showed an initial increase followed by a decrease in the curve (Figure 4).

**Figure 5:**
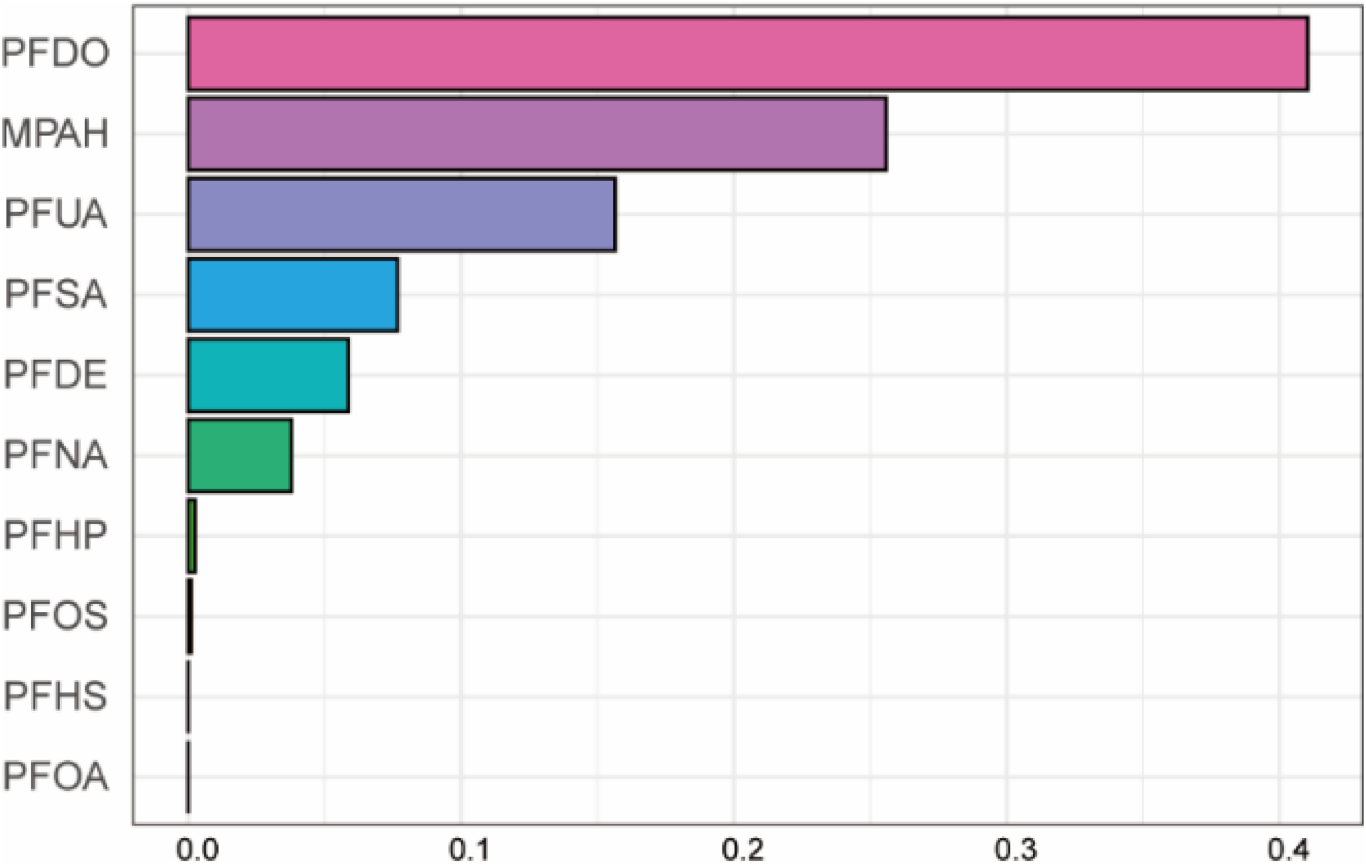
Mixed effects of PFC exposure on CVD using weighted quantile sum (WQS) regression. Adjusted for age, gender, race, educational levels, family income ratio, alcohol, smoking, activities, hypertension, hyperlipidemia, diabetes, BMI, cotinine.

We used the WQS model to discuss the effects of mixed exposure on CVD in both directions and the weights for each PFC. The results showed that there was no significant difference in the bidirectional WQS model (OR, 1.14, 95% CI, 0.90–1.45, *P* = 0.22; OR, 0.98, 95% CI, 0.81–1.18, *P* = 0.82). PFDO, MPAH, PFUA, and PFDE were found top four contributing PFCs and PFDO contributed the most to the combined effect on CVD (Figure 5).

### 3.6 Sensitivity analysis

To support our results, we performed a sensitivity analysis. The information of the sensitivity analysis was presented in Supplementary Figure S3. We adjusted the covariates in different models and the results showed the consistency of the different models.

## 4 Discussion

CVD stands as the foremost cause of mortality worldwide, surpassing all other causes of death annually. The escalating incidence rate of CVD imposes a substantial health and economic burden on many countries. In this population-based retrospective study, we surveyed 4,093 U.S. adults between 2005 and 2012. We comprehensively explored the association between PFCs and CVD using a variety of analytical strategies, including multiple logistic regression, generalized addition models, subgroup analysis, BKMR model, and WQS regression. Our research has found a notable positive association between PFCs and CVD. After adjustment for the included covariates, elevated PFDE, MPAH, and PFUA were positively associated with increased CVD risk. In addition, we uncovered a nonlinear relationship between MPAH and CVD using a generalized additive model. PFUA and PFDE were positively associated CVD in male, non-Hispanic white individuals. In mixed exposure analysis, we observed a trend where the impact of mixed PFC exposure on CVD initially ascended and then declined, with an overall positive effect. Among the PFCs, PFDO contributed most to the effect.

PFCs are synthetic compounds characterized by strong carbon-fluorine bonds (26). They are commonly used in a variety of consumer goods and industrial processes owing to their distinctive properties (16). PFCs can infiltrate the human body through various routes and be measured in human samples such as serum, urine, and even breast milk, posing huge potential risks to human health (27–29). Numerous studies have indicated that PFCs are associated with various human diseases, including elevated uric acid levels, endocrine disorders, oxidative stress, asthma, tumors, hyperuricemia, and immunotoxicity, garnering significant societal attention (30). Studies have pointed out that high levels of PFOA and PFOS may cause an increase in total cholesterol and low-density lipoprotein cholesterol (LDL-C) (31). In another study, higher serum PFOA concentrations were associated with elevated homocysteine levels and hypertension (32). In addition, PFOS has been shown to be associated with insulin resistance (33). Despite of global efforts to phase out and replace PFCs, residual traces of these chemicals in the environment continue to exert prolonged effects on human health.

Recent studies have shown a link between PFCs and the cardiovascular system. After adjusting for traditional cardiovascular confounders, carotid intima-media thickness (CIMT) was significantly increased in the highest quartile of PFOS exposure, suggesting a potential association between PFOS and atherosclerosis (34). Additionally, population studies have demonstrated a positive association between PFOS and the incidence of coronary heart disease, stroke, and peripheral arterial disease (35, 36). In a study conducted in Hebei Province, China, higher levels of PFOA and PFOS and lower levels of PFHS and PFDA were associated with an increased risk of acute coronary syndrome (37). In a cross-sectional study from the United States, perfluoroalkyl substances (PFAS) were found to be associated with the risk of cardiovascular disease (CVD). Specifically, MPAH and PFDO were positively correlated with congestive heart failure, while PFNA, PFDE, and PFUA were positively correlated with coronary heart disease (38). In a diabetes prevention program, pre-diabetic adults with higher levels of PFAS had a higher risk of coronary and thoracic aortic calcification, suggesting that PFAS exposure may be a risk factor for poor cardiovascular health in high-risk populations (39). In the results of another study, exposure to PFOS and PFOA was found to be associated with an increased risk of cardiovascular disease in postmenopausal women (40). In addition, PFAS exposure has been reported for the first time in the pediatric population to affect cardiovascular function and subclinical diseases (41). These epidemiological findings generally align with our results. In this study, we also discovered some perspectives different from previous research. Evidence of the association between PFOA and PFOS with CVD was found before (42), but we did not identify the association. Instead, we observed that high concentrations of PFDE, MPAH, and PFUA were associated with elevated CVD. The heterogeneity of the population may be one reason for this difference. Regulation and policies treating PFCs may lead to increasing external exposure and affect serum internal exposure levels, resulting in different conclusions. In addition, we found a significant nonlinear relationship MPAH between and CVD which does not align with the results of logistic regression. As we know, GAM using smooth function are easy to explain complex nonlinear relationships and more suitable for the effects of environment factors (43). The results of GAM are convincing through cross-validation and comparison of the fitting of models, even though the performance difference between the two models is not significant.

Previous studies have indicated that the concentration of PFCs in serum varies by race/ethnicity and gender (44). Therefore, we considered race and gender as potential interaction factors and conducted subgroup analyses. The results showed that there is an interaction between gender and race in the association between PFCs and CVD. Specifically, non-Hispanic white males exposed to PFCs have a higher risk of occurrence CVD which is consistent with previous findings, possibly due to higher levels of PFCs in their bodies (45–47). The sensitivity of men to PFCs exposure may be related to gender-related physiological factors (hormone levels and fat distribution), and previous studies have found differences in the distribution and metabolic pathways of PFCs in the body between men and women (48). Higher fat stores in men may make it easier for PFUA to accumulate in their bodies, which in turn increases the risk of cardiovascular disease. In addition, there may be differences in the exposure and metabolism of PFCs among different ethnic groups, which may be related to factors such as lifestyle, dietary habits, and environmental exposure levels (49). Especially for non-Hispanic white populations, the relationship between PFUA and PFDE and cardiovascular disease may be more significant due to specific environmental exposure patterns. Our study suggests that non-Hispanic white males may be a susceptible population to the effects of PFCs on CVD, and thus, preventive measures and regular screening could be beneficial for this group.

We believe that the effects of chemicals on outcome are not singular, there may be interactions between them. To study the real relationships between PFCs and CVD, it is necessary to consider the combined effects of chemicals. The results of the BKMR model suggested that higher mixed PFC exposure is related to elevated CVD which is consistent with the results of single exposure analyses. The outcomes of the WQS regression suggest that PFDO, MPAH, PFUA and PFDE are the top 4 PFCs contributing to the joint effect on CVD. Among them, the results for MPAH, PFUA, and PFDE are consistent with those of logistic regression. Although no significant relationship was observed between PFDO and CVD, PFDO was found to contribute most to the occurrence of CVD in the WQS model. This discrepancy might be due to PFDO's effects being influenced by other chemicals, thereby diluting its individual impact. However, the WQS model evaluates the contribution of PFCs comprehensively by considering the combined effects of multiple chemicals, accounting for nonlinear relationships, reducing collinearity issues, improving statistical power, and assigning appropriate weights (50). Therefore, even if PFDO is not significant in a single-exposure analysis, it may still contribute the most in the WQS model.

Numerous studies have confirmed the role of inflammation in the association between PFCs and CVD. In a Swedish study, both experimental and epidemiological evidence suggested that PFAS exposure was linked to changes in protein levels associated with inflammation, metabolism, and cardiovascular disease in middle-aged adults (38). Specifically, PFOA, PFOS and PFHxS were found to significantly overlap with proteins such as EGFR, PON3, uPAR and RETN, which are known to be associated with markers of inflammation such as IL-6 in white blood cells. Furthermore, animal experiments have shown that PFOS can accelerate the progression of atherosclerosis in ApoE - -mice, possibly through the activation of macrophage NF-κB, leading to M1 polarization and inflammation (51). In addition to inflammation, some biological studies have provided evidence of the impact of PFCs on cardiovascular disease. Some studies have shown that exposure to PFOS is directly related to atherosclerosis, and indirectly related to atherosclerosis through DNA methylation (52). Our study provides evidence supporting an association between serum PFCs and CVD, thereby contributing to further mechanistic research in this area.

Our study presents several notable strengths. To our knowledge, it is the first investigation to explore the relationship between both individual and combined exposures to PFCs and CVD. Through meticulous adjustment for confounding variables and subgroup analyses, we have discerned a positive correlation between PFDE, PFUA, and CVD. Moreover, our analysis of mixed exposures to PFCs revealed a trend wherein the impact initially increases before decreasing, ultimately exerting a net-promoting effect on CVD.

However, our study is not without limitations. Primarily, it relies on data from the NHANES database, which is inherently cross-sectional, unlikely to infer causal relationships between environmental exposure and outcomes, warranting further prospective investigations. Additionally, the possibility of unmeasured confounding factors influencing our results cannot be discounted, potentially introducing bias. Lastly, the precise pathogenic mechanisms of PFCs remain elusive and require substantiation through further experimental research.

## 5 Conclusions

In summary, our investigation reveals a noteworthy positive correlation between serum PFDE, MPAH, and PFUA levels with CVD. We also found interactions between PFUA and gender and race related to CVD. Moreover, a general positive correlation exists between mixed exposure to PFCs and CVD, with PFDO contributing the most among them. Our study provided important evidence for probing the impact of PFCs on CVD. However, further research is imperative to elucidate the causal relationship between these associations.

## Data Availability

Publicly available datasets were analyzed in this study. This data can be found here: https://wwwn.cdc.gov/nchs/nhanes/.
