# Supplementary material for: Exposure to specific polyfluoroalkyl chemicals is associated with cardiovascular disease in US adults: a population-based study
Source: Front Cardiovasc Med. 2025 Jan 9;11:1487956. doi: 10.3389/fcvm.2024.1487956 (PMC11754394; doi:10.3389/fcvm.2024.1487956)
Supplement: Supplementary file 1 [file Datasheet1.docx]

**Supplementary Materials**

**Exposure to specific polyfluoroalkyl chemicals is associated with cardiovascular disease in US adults: A population-based study**

Wenwen Xiao ^a 1^, Guojin Jian ^b 1^, Fei Ma ^a^, Hong Li ^a^, Xiaohong Yang ^a^, Hengyang Zhang ^a^, Yongping Cao ^a *^

a. Eastern Theater Command Centers for Disease Control and Prevention, Nanjing, China

b. Department of Cardiology, PLA Joint Logistic Support Force 902 Hospital, Bengbu, China

^1^ These authors contributed equally.

*Corresponding Author, Yongping Cao (caoyongping_1970@126.com).

**Contents**

**Table S1.** The limits of detection and detection rates of PFCs.

**Table S2.** Characteristics of the study population.

**Table S3.** Subgroup analysis for the associations between PFCs and CVD in relation to education.

**Table S4.** Subgroup analysis for the associations between PFCs and CVD in relation to age.

**Table S5.** Subgroup analysis for the associations between PFCs and CVD in relation to alcohol.

**Table S6.** Subgroup analysis for the associations between PFCs and CVD in relation to smoke.

**Table S7.** Subgroup analysis for the associations between PFCs and CVD in relation to smoke.

**Table S8.** Subgroup analysis for the associations between PFCs and CVD in relation to diabetes.

**Table S9.** Subgroup analysis for the associations between PFCs and CVD in relation to hypertension.

**Table S10.** Subgroup analysis for the associations between PFCs and CVD in relation to hyperlipidemia.

**Table S11.** Subgroup analysis for the associations between PFCs and CVD in relation to BMI.

**Table S12.** WQS model to estimate the associations between WQS index and CVD.

**Table S5.** WQS model to estimate the associations between WQS index and CVD.

**Fig. S1.** Spearman correlations among 10 PFCs in the population (N =4093), NHANES, [USA](https://www.sciencedirect.com/topics/earth-and-planetary-sciences/united-states-of-america), 2005–2012.

**Fig. S2.** BKMR analysis on the PFCs exposure and CVD. The impact of a single PFC, when the single PFC is in the 75th percentile and the 25th percentile, on the potential continuous outcome of CVD, while all PFCs are in the 25th, 50th, or 75th percentile. "Est" is defined as the association between a single PFC and the potential continuous outcome.

**Fig. S3.** Sensitivity analysis performed by changing the adjusted covariates. Model1: Unadjusted. Model 2: Adjusted for age, gender, race, educational levels, family income ratio. Model 3: Adjusted for age, gender, race, educational levels, family income ratio, alcohol, smoking, activities, hypertension, hyperlipidemia, diabetes, BMI, cotinine.

**Table S1.** The limits of detection and detection rates of PFCs.

| Variables | LOD (ng/mL) |  | Detection rate (%) |
| --- | --- | --- | --- |
| PFOA | 0.1 |  | 99.68% |
| PFOS | 0.2 |  | 99.73% |
| PFDE | 0.2 |  | 83.19% |
| PFHS | 0.1 |  | 98.68% |
| MPAH | 0.2 |  | 70.80% |
| PFNA | 0.1 |  | 99.43% |
| PFHP | 0.4 |  | 12.73% |
| PFSA | 0.1 |  | 7.180% |
| PFDO | 0.2 |  | 5.70% |
| PFUA | 0.2 |  | 54.70% |

PFCs, polyfluoroalkyl chemicals; LOD, limit of detection; PFOA, perfluorooctanoic acid; FPOS, perfluorooctane sulfonate acid; PFHS, perfluorohexane; sulfonate acid; MPAH, 2-(N-methyl perfluorooctane sulfonate) acid; PFDE, perfluorodecanoic acid; PFHP, perfluoroheptanoic acid; PFNA, perfluorononanoic acid; PFSA perfluorooctane sulfonate; PFUA, perfluoroundecanoic acid; PFDO, perfluorododecanoic acid.

**Table S2.** Characteristics of the study population.

| Variables | Non-CVD (n=3632) | CVD (n=461) | P value |
| --- | --- | --- | --- |
| PFOA (ng/mL) | 3.87 (3.32) | 4.32 (2.92) | <0.01 |
| PFOS (ng/mL) | 15.75 (15.72) | 21.82 (24.43) | <0.01 |
| PFDE (ng/mL) | 2.38 (3.13) | 2.55 (3.05) | 0.27 |
| PFHS (ng/mL) | 0.38 (0.58) | 0.56 (0.75) | <0.01 |
| MPAH (ng/mL) | 0.40 (0.6) | 0.48 (0.6) | <0.01 |
| PFNA (ng/mL) | 0.11 (0.08) | 0.11 (0.05) | 0.24 |
| PFHP (ng/mL) | 0.20 (0.27) | 0.21 (0.16) | 0.45 |
| PFSA (ng/mL) | 1.41 (1.22) | 1.72 (1.77) | <0.01 |
| PFDO (ng/mL) | 0.08 (0.10) | 0.09 (0.10) | 0.02 |
| PFUA (ng/mL) | 0.28 (0.46) | 0.35 (0.62) | <0.01 |

CVD, cardiovascular disease; PFOA, perfluorooctanoic acid; FPOS, perfluorooctane sulfonate acid; PFHS, perfluorohexane; sulfonate acid; MPAH, 2- (N-methyl perfluorooctane sulfonate) acid; PFDE, perfluorodecanoic acid; PFHP, perfluoroheptanoic acid; PFNA, perfluorononanoic acid; PFSA perfluorooctane sulfonate; PFUA, perfluoroundecanoic acid; PFDO, perfluorododecanoic acid.

**Table S3.** Subgroup analysis for the associations between PFCs and CVD in relation to education.

| Education | High school and below | | | Other | | |  |
| --- | --- | --- | --- | --- | --- | --- | --- |
|  | OR | CI | P value | OR | CI | P value | P-int |
| PFOA | 0.9 | -0.29-0.09 | 0.3 | 1.03 | 0.81-1.32 | 0.81 | 0.37 |
| PFOS | 0.99 | 0.85-1.16 | 0.92 | 1.08 | 0.88-1.34 | 0.44 | 0.2 |
| PFDE | 1.15 | 0.96-1.37 | 0.12 | 1.22 | 0.97-1.52 | 0.09 | 0.34 |
| PFHS | 0.86 | 0.73-1.01 | 0.07 | 0.94 | 0.76-1.15 | 0.52 | 0.33 |
| MPAH | 1.19 | 1.03-1.38 | 0.02^*^ | 1.1 | 0.93-1.32 | 0.27 | 0.98 |
| PFNA | 1.15 | 0.94-1.40 | 0.17 | 1.01 | 0.79-1.30 | 0.91 | 0.71 |
| PFHP | 0.93 | 0.76-1.13 | 0.45 | 1.28 | 1.02-1.62 | 0.04 | 0.04^*^ |
| PFSA | 1.21 | 0.82-1.75 | 0.32 | 1.31 | 0.80-2.11 | 0.27 | 0.68 |
| PFDO | 0.97 | 0.68-1.37 | 0.85 | 1.99 | 1.28-3.11 | <0.01^**^ | <0.01^**^ |
| PFUA | 1.14 | 0.95-1.36 | 0.15 | 1.37 | 1.09-1.72 | <0.01^**^ | 0.06 |

Analysis was adjusted for gender, age, race, education level, family income ratio, smoking, drinking, physical activity, diabetes, hypertension, hyperlipidemia, BMI, cotinine. * P < 0.05, * * P < 0.01. PFCs, polyfluoroalkyl chemicals; CVD, cardiovascular disease; OR, odds ratio; 95% CI, 95 % confidence interval; P-int, p for interaction; PFOA, perfluorooctanoic acid; FPOS, perfluorooctane sulfonate acid; PFHS, perfluorohexane; sulfonate acid; MPAH, 2- (N-methyl perfluorooctane sulfonate) acid; PFDE, perfluorodecanoic acid; PFHP, perfluoroheptanoic acid; PFNA, perfluorononanoic acid; PFSA perfluorooctane sulfonate; PFUA, perfluoroundecanoic acid; PFDO, perfluorododecanoic acid.

**Table S4.** Subgroup analysis for the associations between PFCs and CVD in relation to age.

| Age | <55 | | | ≥55 | | |  |
| --- | --- | --- | --- | --- | --- | --- | --- |
|  | OR | CI | P value | OR | CI | P value | P-int |
| PFOA | 0.93 | 0.64-1.38 | 0.7 | 1.04 | 0.88-1.22 | 0.68 | 0.19 |
| PFOS | 0.93 | 0.68-1.30 | 0.67 | 1.15 | 1.00-1.32 | 0.05^*^ | 0.11 |
| PFDE | 0.94 | 0.63-1.39 | 0.77 | 1.24 | 1.07-1.43 | <0.01^**^ | 0.15 |
| PFHS | 0.84 | 0.61-1.14 | 0.26 | 0.99 | 0.86-1.13 | 0.85 | 0.16 |
| MPAH | 1.17 | 0.85-1.60 | 0.34 | 1.24 | 1.10-1.40 | <0.01^**^ | 0.84 |
| PFNA | 1.1 | 0.72-1.72 | 0.66 | 1.13 | 0.96-1.33 | 0.15 | 0.93 |
| PFHP | 1.17 | 0.79-1.72 | 0.42 | 1.08 | 0.92-1.26 | 0.37 | 0.13 |
| PFSA | 2.5 | 0.51-1.29 | 0.26 | 1.61 | 1.18-2.19 | <0.01^**^ | 0.15 |
| PFDO | 0.75 | 0.37-1.41 | 0.4 | 1.45 | 1.08-1.95 | 0.01^*^ | 0.53 |
| PFUA | 1.11 | 0.74-1.61 | 0.59 | 1.29 | 1.11-1.50 | <0.01^**^ | 0.47 |

Analysis was adjusted for gender, age, race, education level, family income ratio, smoking, drinking, physical activity, diabetes, hypertension, hyperlipidemia, BMI, cotinine. * P < 0.05, * * P < 0.01. PFCs, polyfluoroalkyl chemicals; CVD, cardiovascular disease; OR, odds ratio; 95% CI, 95 % confidence interval; P-int, p for interaction; PFOA, perfluorooctanoic acid; FPOS, perfluorooctane sulfonate acid; PFHS, perfluorohexane; sulfonate acid; MPAH, 2- (N-methyl perfluorooctane sulfonate) acid; PFDE, perfluorodecanoic acid; PFHP, perfluoroheptanoic acid; PFNA, perfluorononanoic acid; PFSA perfluorooctane sulfonate; PFUA, perfluoroundecanoic acid; PFDO, perfluorododecanoic acid.

**Table S5.** Subgroup analysis for the associations between PFCs and CVD in relation to alcohol.

| Alcohol | Yes | | | No | | |  |
| --- | --- | --- | --- | --- | --- | --- | --- |
|  | OR | CI | P value | OR | CI | P value | P-int |
| PFOA | 1.03 | 0.85-1.25 | 0.80 | 0.84 | 0.65-1.08 | 0.17 | 0.36 |
| PFOS | 1.08 | 0.93-1.26 | 0.33 | 0.90 | 0.73-1.12 | 0.35 | 0.3 |
| PFDE | 1.18 | 1.00-1.39 | 0.05^*^ | 1.14 | 0.89-1.46 | 0.29 | 0.62 |
| PFHS | 0.89 | 0.76-1.04 | 0.13 | 0.87 | 0.70-1.09 | 0.22 | 0.71 |
| MPAH | 1.12 | 0.98-1.29 | 0.09 | 1.21 | 0.99-1.48 | 0.06 | 0.49 |
| PFNA | 1.19 | 0.98-1.44 | 0.08 | 0.95 | 0.73-1.23 | 0.67 | 0.14 |
| PFHP | 1.15 | 0.96-1.37 | 0.14 | 0.88 | 0.67-1.15 | 0.35 | 0.16 |
| PFSA | 1.23 | 0.87-1.70 | 0.22 | 1.42 | 0.70-2.81 | 0.31 | 0.54 |
| PFDO | 1.33 | 0.96-1.83 | 0.08 | 1.07 | 0.63-1.79 | 0.79 | 0.44 |
| PFUA | 1.21 | 1.02-1.42 | 0.03^*^ | 1.24 | 0.95-1.61 | 0.11 | 0.9 |

Analysis was adjusted for gender, age, race, education level, family income ratio, smoking, drinking, physical activity, diabetes, hypertension, hyperlipidemia, BMI, cotinine. * P < 0.05, * * P < 0.01. PFCs, polyfluoroalkyl chemicals; CVD, cardiovascular disease; OR, odds ratio; 95% CI, 95 % confidence interval; P-int, p for interaction; PFOA, perfluorooctanoic acid; FPOS, perfluorooctane sulfonate acid; PFHS, perfluorohexane; sulfonate acid; MPAH, 2- (N-methyl perfluorooctane sulfonate) acid; PFDE, perfluorodecanoic acid; PFHP, perfluoroheptanoic acid; PFNA, perfluorononanoic acid; PFSA perfluorooctane sulfonate; PFUA, perfluoroundecanoic acid; PFDO, perfluorododecanoic acid.

**Table S6.** Subgroup analysis for the associations between PFCs and CVD in relation to smoke.

| Smoke | Yes | | | No | | |  |
| --- | --- | --- | --- | --- | --- | --- | --- |
|  | OR | CI | P value | OR | CI | P value | P-int |
| PFOA | 1.04 | 0.85-1.27 | 0.73 | 0.86 | 0.68-1.09 | 0.2 | 0.39 |
| PFOS | 1.06 | 0.91-1.24 | 0.47 | 0.96 | 0.79-1.19 | 0.73 | 0.79 |
| PFDE | 1.22 | 1.02-1.45 | 0.03^*^ | 1.11 | 0.88-1.39 | 0.38 | 0.52 |
| PFHS | 0.93 | 0.79-1.09 | 0.36 | 0.84 | 0.69-1.03 | 0.09 | 0.76 |
| MPAH | 1.13 | 0.98-1.31 | 0.10 | 1.18 | 1.00-1.41 | 0.06 | 0.44 |
| PFNA | 1.19 | 0.98-1.45 | 0.08 | 0.97 | 0.76-1.24 | 0.8 | 0.25 |
| PFHP | 1.16 | 0.96-1.40 | 0.12 | 0.92 | 0.72-1.18 | 0.52 | 0.16 |
| PFSA | 1.28 | 0.89-1.82 | 0.17 | 1.28 | 0.73-2.15 | 0.37 | 0.84 |
| PFDO | 1.44 | 1.02-2.02 | 0.04^*^ | 1.04 | 0.66-1.63 | 0.86 | 0.25 |
| PFUA | 1.21 | 1.01-1.44 | 0.04^*^ | 1.25 | 1.00-.1.55 | 0.05* | 0.76 |

Analysis was adjusted for gender, age, race, education level, family income ratio, smoking, drinking, physical activity, diabetes, hypertension, hyperlipidemia, BMI, cotinine. * P < 0.05, * * P < 0.01. PFCs, polyfluoroalkyl chemicals; CVD, cardiovascular disease; OR, odds ratio; 95% CI, 95 % confidence interval; P-int, p for interaction; PFOA, perfluorooctanoic acid; FPOS, perfluorooctane sulfonate acid; PFHS, perfluorohexane; sulfonate acid; MPAH, 2- (N-methyl perfluorooctane sulfonate) acid; PFDE, perfluorodecanoic acid; PFHP, perfluoroheptanoic acid; PFNA, perfluorononanoic acid; PFSA perfluorooctane sulfonate; PFUA, perfluoroundecanoic acid; PFDO, perfluorododecanoic acid.

**Table S7.** Subgroup analysis for the associations between PFCs and CVD in relation to smoke.

| Activities | Yes | | | No | | |  |
| --- | --- | --- | --- | --- | --- | --- | --- |
|  | OR | CI | P value | OR | CI | P value | P-int |
| PFOA | 1.32 | 0.89-2.04 | 0.19 | 0.91 | 0.77-1.07 | 0.26 | 0.18 |
| PFOS | 1.20 | 0.85-1.73 | 0.31 | 0.99 | 0.86-1.13 | 0.84 | 0.37 |
| PFDE | 1.26 | 0.87-1.81 | 0.22 | 1.16 | 1.00-1.35 | 0.06 | 0.52 |
| PFHS | 0.91 | 0.66-1.26 | 0.57 | 0.89 | 0.77-1.02 | 0.09 | 0.98 |
| MPAH | 1.31 | 1.00-1.71 | 0.05^*^ | 1.11 | 0.98-1.26 | 0.09 | 0.65 |
| PFNA | 1.06 | 0.70-1.62 | 0.79 | 1.09 | 0.93-1.29 | 0.29 | 0.77 |
| PFHP | 0.99 | 0.68-1.44 | 0.96 | 1.09 | 0.92-1.28 | 0.33 | 0.4 |
| PFSA | 1.26 | 0.56-2.63 | 0.55 | 1.26 | 0.90-1.74 | 0.16 | 0.7 |
| PFDO | 1.18 | 0.58-2.36 | 0.64 | 1.30 | 0.96-1.74 | 0.09 | 0.65 |
| PFUA | 1.27 | 0.87-1.84 | 0.21 | 1.20 | 1.03-1.40 | 0.02* | 0.72 |

Analysis was adjusted for gender, age, race, education level, family income ratio, smoking, drinking, physical activity, diabetes, hypertension, hyperlipidemia, BMI, cotinine. * P < 0.05, * * P < 0.01. PFCs, polyfluoroalkyl chemicals; CVD, cardiovascular disease; OR, odds ratio; 95% CI, 95 % confidence interval; P-int, p for interaction; PFOA, perfluorooctanoic acid; FPOS, perfluorooctane sulfonate acid; PFHS, perfluorohexane; sulfonate acid; MPAH, 2- (N-methyl perfluorooctane sulfonate) acid; PFDE, perfluorodecanoic acid; PFHP, perfluoroheptanoic acid; PFNA, perfluorononanoic acid; PFSA perfluorooctane sulfonate; PFUA, perfluoroundecanoic acid; PFDO, perfluorododecanoic acid.

**Table S8.** Subgroup analysis for the associations between PFCs and CVD in relation to diabetes.

| Diabetes | Yes | | | No | | |  |
| --- | --- | --- | --- | --- | --- | --- | --- |
|  | OR | CI | P value | OR | CI | P value | P-int |
| PFOA | 0.99 | 0.76-1.29 | 0.92 | 0.94 | 0.78-1.13 | 0.51 | 0.96 |
| PFOS | 1.07 | 0.86-1.35 | 0.56 | 0.99 | 0.85-1.16 | 0.94 | 0.89 |
| PFDE | 1.53 | 1.18-2.00 | <0.01^**^ | 1.05 | 0.89-1.24 | 0.58 | 0.06 |
| PFHS | 0.93 | 0.73-1.18 | 0.56 | 0.87 | 0.75-1.02 | 0.08 | 0.97 |
| MPAH | 1.20 | 0.98-1.48 | 0.07 | 1.12 | 0.98-1.28 | 0.10 | 0.83 |
| PFNA | 1.19 | 0.90-1.59 | 0.22 | 1.05 | 0.87-1.27 | 0.59 | 0.66 |
| PFHP | 1.22 | 0.92-1.61 | 0.16 | 1.00 | 0.83-1.20 | 0.99 | 0.32 |
| PFSA | 1.85 | 0.88-3.96 | 0.10 | 1.15 | 0.81-1.60 | 0.41 | 0.31 |
| PFDO | 2.09 | 1.27-3.47 | <0.01^**^ | 1.01 | 0.72-1.40 | 0.95 | 0.02^*^ |
| PFUA | 1.41 | 1.08-1.85 | 0.01^*^ | 1.14 | 0.97-1.35 | 0.11 | 0.42 |

Analysis was adjusted for gender, age, race, education level, family income ratio, smoking, drinking, physical activity, diabetes, hypertension, hyperlipidemia, BMI, cotinine. * P < 0.05, * * P < 0.01. PFCs, polyfluoroalkyl chemicals; CVD, cardiovascular disease; OR, odds ratio; 95% CI, 95 % confidence interval; P-int, p for interaction; PFOA, perfluorooctanoic acid; FPOS, perfluorooctane sulfonate acid; PFHS, perfluorohexane; sulfonate acid; MPAH, 2- (N-methyl perfluorooctane sulfonate) acid; PFDE, perfluorodecanoic acid; PFHP, perfluoroheptanoic acid; PFNA, perfluorononanoic acid; PFSA perfluorooctane sulfonate; PFUA, perfluoroundecanoic acid; PFDO, perfluorododecanoic acid.

**Table S9.** Subgroup analysis for the associations between PFCs and CVD in relation to hypertension.

| Hypertension | Yes | | | No | | |  |
| --- | --- | --- | --- | --- | --- | --- | --- |
|  | OR | CI | P value | OR | CI | P value | P-int |
| PFOA | 0.90 | 0.76-1.07 | 0.22 | 1.20 | 0.87-1.70 | 0.27 | 0.15 |
| PFOS | 1.00 | 0.87-1.16 | 0.97 | 1.08 | 0.82-1.42 | 0.58 | 0.29 |
| PFDE | 1.17 | 1.00-1.38 | 0.05 | 1.16 | 0.88-1.51 | 0.29 | 0.93 |
| PFHS | 0.88 | 0.76-1.02 | 0.09 | 0.91 | 0.71-1.19 | 0.51 | 0.51 |
| MPAH | 1.17 | 0.71-1.19 | 0.02 | 1.09 | 0.88-1.36 | 0.41 | 0.77 |
| PFNA | 1.13 | 0.95-1.34 | 0.18 | 1.03 | 0.74-1.43 | 0.88 | 0.66 |
| PFHP | 0.98 | 0.82-1.17 | 0.84 | 1.26 | 0.95-1.69 | 0.11 | 0.12 |
| PFSA | 1.38 | 0.92-2.05 | 0.11 | 1.22 | 0.73-1.91 | 0.41 | 0.64 |
| PFDO | 1.11 | 0.81-1.54 | 0.51 | 1.67 | 1.00-2.75 | 0.05* | 0.2 |
| PFUA | 1.21 | 1.03-1.42 | 0.02* | 1.27 | 0.96-1.68 | 0.09 | 0.75 |

Analysis was adjusted for gender, age, race, education level, family income ratio, smoking, drinking, physical activity, diabetes, hypertension, hyperlipidemia, BMI, cotinine. * P < 0.05, * * P < 0.01. PFCs, polyfluoroalkyl chemicals; CVD, cardiovascular disease; OR, odds ratio; 95% CI, 95 % confidence interval; P-int, p for interaction; PFOA, perfluorooctanoic acid; FPOS, perfluorooctane sulfonate acid; PFHS, perfluorohexane; sulfonate acid; MPAH, 2- (N-methyl perfluorooctane sulfonate) acid; PFDE, perfluorodecanoic acid; PFHP, perfluoroheptanoic acid; PFNA, perfluorononanoic acid; PFSA perfluorooctane sulfonate; PFUA, perfluoroundecanoic acid; PFDO, perfluorododecanoic acid.

**Table S10.** Subgroup analysis for the associations between PFCs and CVD in relation to hyperlipidemia.

| Hyperlipidemia | Yes | | | No | | |  |
| --- | --- | --- | --- | --- | --- | --- | --- |
|  | OR | CI | P value | OR | CI | P value | P-int |
| PFOA | 0.92 | 0.76-1.13 | 0.43 | 0.99 | 0.78-1.27 | 0.93 | 0.88 |
| PFOS | 0.92 | 0.79-1.08 | 0.31 | 1.18 | 0.95-1.46 | 0.14 | 0.08 |
| PFDE | 1.16 | 0.96-1.39 | 0.12 | 1.20 | 0.97-1.48 | 0.10 | 0.61 |
| PFHS | 0.87 | 0.74-1.03 | 0.11 | 0.94 | 0.76-1.15 | 0.52 | 0.82 |
| MPAH | 1.09 | 0.94-1.26 | 0.24 | 1.25 | 1.04-1.50 | 0.02^*^ | 0.39 |
| PFNA | 1.05 | 0.86-1.28 | 0.63 | 1.16 | 0.91-1.50 | 0.24 | 0.42 |
| PFHP | 1.08 | 0.89-1.31 | 0.45 | 1.03 | 0.81-1.30 | 0.83 | 0.63 |
| PFSA | 1.17 | 0.78-1.71 | 0.44 | 1.45 | 0.89-2.30 | 0.12 | 0.72 |
| PFDO | 1.20 | 0.85-1.70 | 0.29 | 1.32 | 0.84-2.08 | 0.22 | 0.78 |
| PFUA | 1.22 | 1.02-1.47 | 0.03^*^ | 1.24 | 0.99-1.54 | 0.06 | 0.52 |

Analysis was adjusted for gender, age, race, education level, family income ratio, smoking, drinking, physical activity, diabetes, hypertension, hyperlipidemia, BMI, cotinine. * P < 0.05, * * P < 0.01. PFCs, polyfluoroalkyl chemicals; CVD, cardiovascular disease; OR, odds ratio; 95% CI, 95 % confidence interval; P-int, p for interaction; PFOA, perfluorooctanoic acid; FPOS, perfluorooctane sulfonate acid; PFHS, perfluorohexane; sulfonate acid; MPAH, 2- (N-methyl perfluorooctane sulfonate) acid; PFDE, perfluorodecanoic acid; PFHP, perfluoroheptanoic acid; PFNA, perfluorononanoic acid; PFSA perfluorooctane sulfonate; PFUA, perfluoroundecanoic acid; PFDO, perfluorododecanoic acid.

**Table S11.** Subgroup analysis for the associations between PFCs and CVD in relation to BMI.

| BMI | <25 | | | ≥25 | | |  |
| --- | --- | --- | --- | --- | --- | --- | --- |
|  | OR | CI | P value | OR | CI | P value | P-int |
| PFOA | 0.93 | 0.68-1.27 | 0.63 | 1 | 0.84-1.20 | 1 | 0.54 |
| PFOS | 1.14 | 0.88-1.50 | 0.33 | 1 | 0.87-1.16 | 0.97 | 0.23 |
| PFDE | 1.26 | 0.95-1.66 | 0.11 | 1.13 | 0.97-1.33 | 0.12 | 0.32 |
| PFHS | 0.91 | 0.70-1.18 | 0.46 | 0.9 | 0.78-1.05 | 0.17 | 0.83 |
| MPAH | 1.08 | 0.86-1.36 | 0.51 | 1.19 | 1.05-1.35 | <0.01^**^ | 0.31 |
| PFNA | 1.1 | 0.80-1.52 | 0.57 | 1.12 | 0.94-1.33 | 0.22 | 0.95 |
| PFHP | 1.21 | 0.87-1.69 | 0.26 | 1.02 | 0.86-1.21 | 0.79 | 0.48 |
| PFSA | 1.6 | 0.89-2.72 | 0.1 | 1.16 | 0.80-1.65 | 0.41 | 0.46 |
| PFDO | 1.77 | 0.97-3.22 | 0.06 | 1.16 | 0.85-1.57 | 0.35 | 0.36 |
| PFUA | 1.17 | 0.87-1.55 | 0.29 | 1.21 | 1.03-1.43 | 0.02^*^ | 0.78 |

Analysis was adjusted for gender, age, race, education level, family income ratio, smoking, drinking, physical activity, diabetes, hypertension, hyperlipidemia, BMI, cotinine. * P < 0.05, * * P < 0.01. PFCs, polyfluoroalkyl chemicals; CVD, cardiovascular disease; OR, odds ratio; 95% CI, 95 % confidence interval; P-int, p for interaction; PFOA, perfluorooctanoic acid; FPOS, perfluorooctane sulfonate acid; PFHS, perfluorohexane; sulfonate acid; MPAH, 2- (N-methyl perfluorooctane sulfonate) acid; PFDE, perfluorodecanoic acid; PFHP, perfluoroheptanoic acid; PFNA, perfluorononanoic acid; PFSA perfluorooctane sulfonate; PFUA, perfluoroundecanoic acid; PFDO, perfluorododecanoic acid.

**Table S12.** WQS model to estimate the associations between WQS index and CVD.

| CVD | OR | 95% CI | P value |
| --- | --- | --- | --- |
| Positive | 1.14 | 0.90-1.45 | 0.22 |
| Negative | 0.98 | 0.81-1.18 | 0.82 |

OR: odds ratio; CI: confidence interval; OR estimates represent the odds ratios of general or abdominal obesity when the WQS index was increased by one quartile. Model was adjusted for age, gender, race, educational levels, family income ratio, alcohol, smoking, activities, hypertension, hyperlipidemia, diabetes, BMI, cotinine.

**
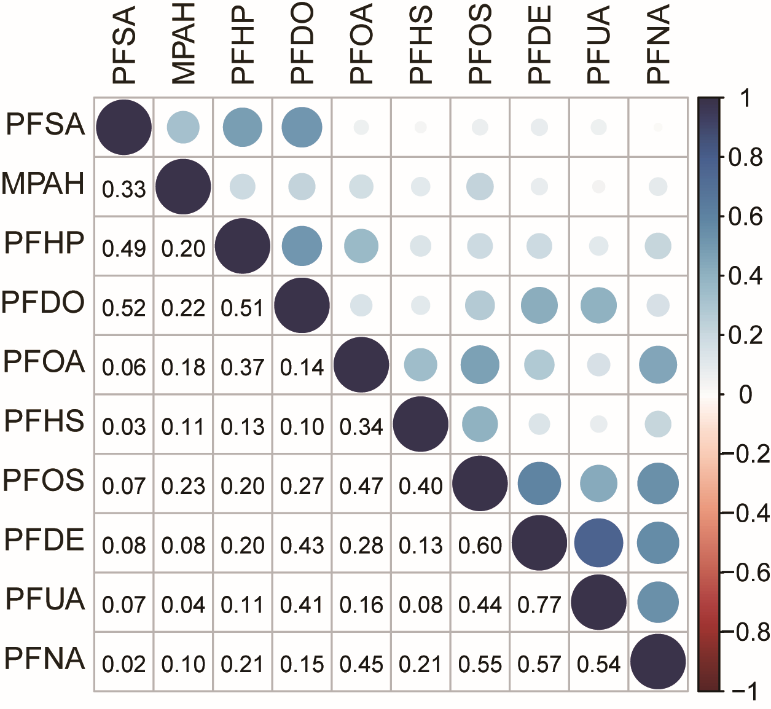
**

**Fig. S1.** Spearman correlations among 10 PFCs in the population (N =4093), NHANES, [USA](https://www.sciencedirect.com/topics/earth-and-planetary-sciences/united-states-of-america), 2005–2012.


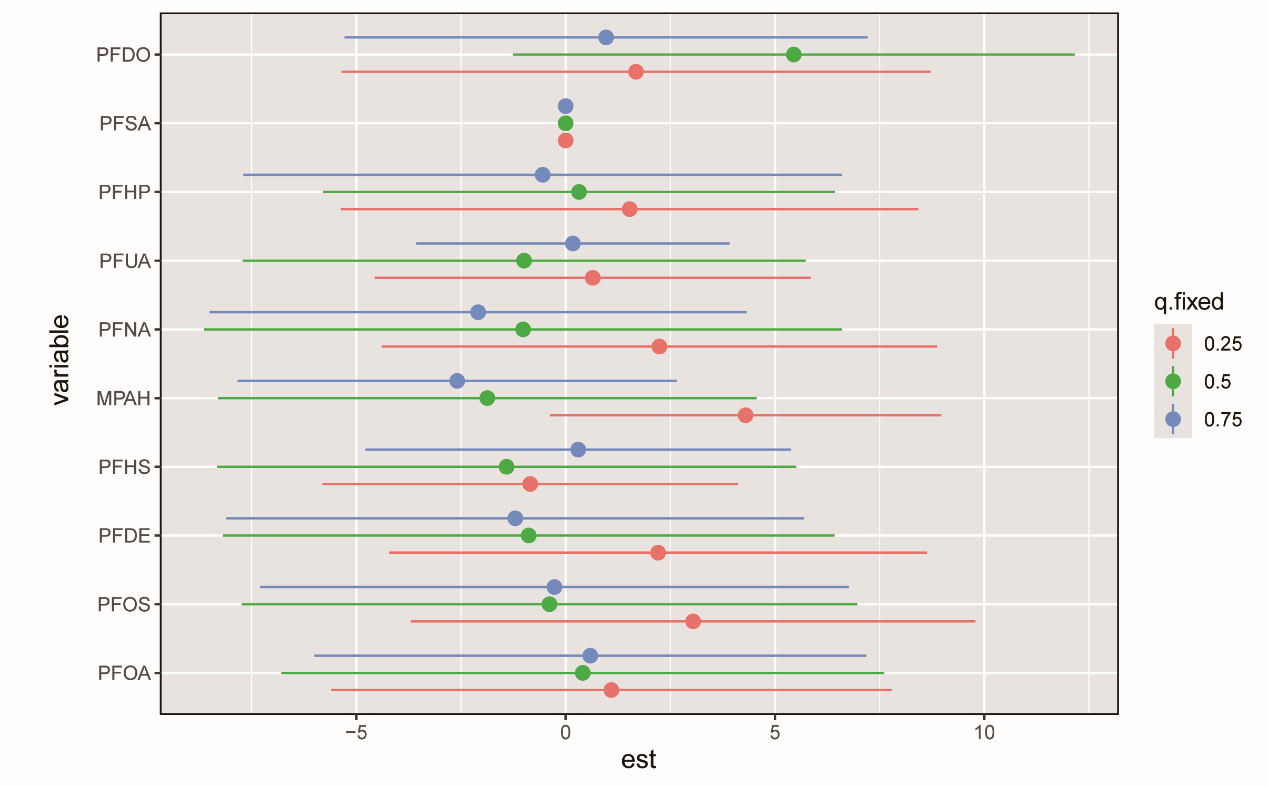


**Fig. S2.** BKMR analysis on the PFCs exposure and CVD. The impact of a single PFC, when the single PFC is in the 75th percentile and the 25th percentile, on the potential continuous outcome of CVD, while all PFCs are in the 25th, 50th, or 75th percentile. "Est" is defined as the association between a single PFC and the potential continuous outcome.


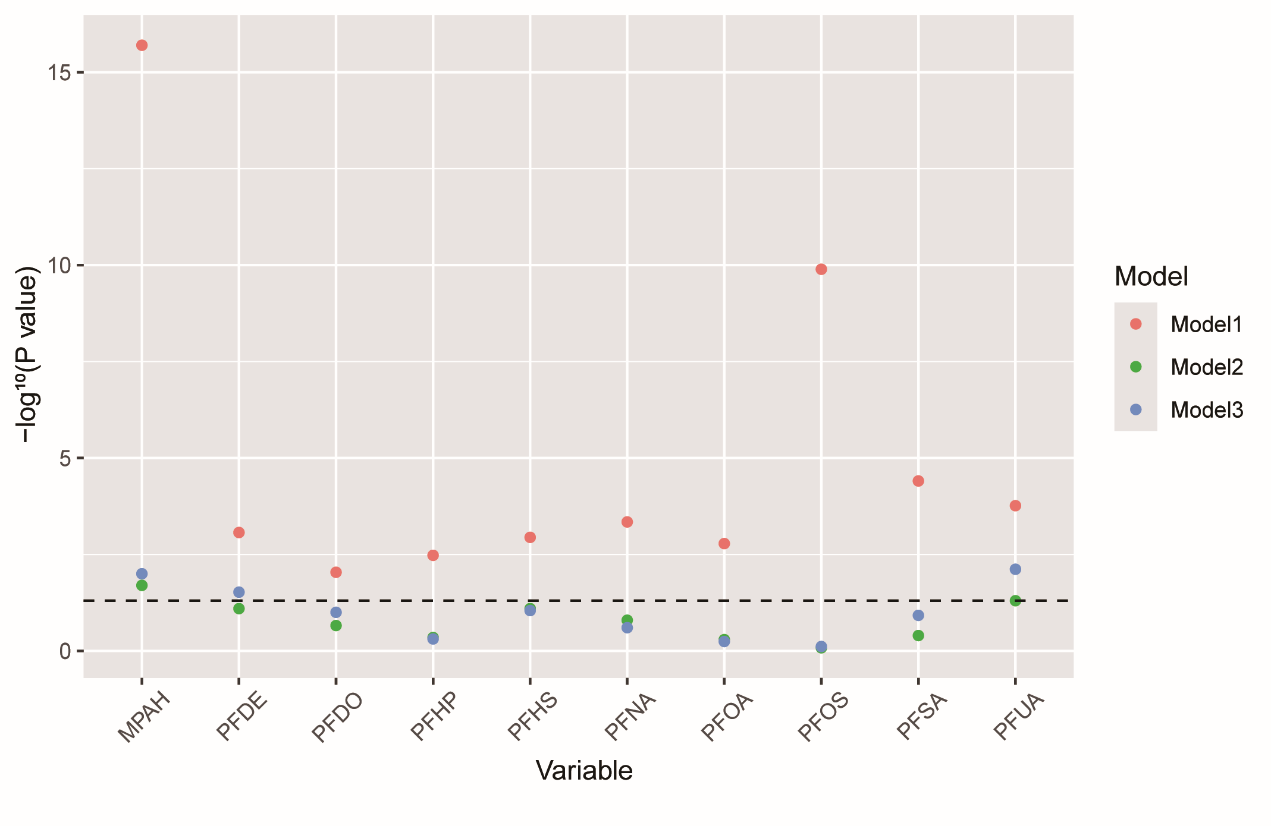
**Fig. S3.** Sensitivity analysis performed by changing the adjusted covariates. Model 1: Unadjusted. Model 2: Adjusted for age, gender, race, educational levels, family income ratio. Model 3: Adjusted for age, gender, race, educational levels, family income ratio, alcohol, smoking, activities, hypertension, hyperlipidemia, diabetes, BMI, cotinine.
